# Psychopathy to Altruism: Neurobiology of the Selfish–Selfless Spectrum

**DOI:** 10.3389/fpsyg.2018.00575

**Published:** 2018-04-19

**Authors:** James W. H. Sonne, Don M. Gash

**Affiliations:** ^1^Department of Health Professions, University of Central Florida, Orlando, FL, United States; ^2^Department of Neuroscience, University of Kentucky, Lexington, KY, United States

**Keywords:** heredity, social, cultural, genetic, neural circuitry, emotions, empathy, compassion

## Abstract

The age-old philosophical, biological, and social debate over the basic nature of humans as being “universally selfish” or “universally good” continues today highlighting sharply divergent views of natural social order. Here we analyze advances in biology, genetics and neuroscience increasing our understanding of the evolution, features and neurocircuitry of the human brain underlying behavior in the selfish–selfless spectrum. First, we examine evolutionary pressures for selection of altruistic traits in species with protracted periods of dependence on parents and communities for subsistence and acquisition of learned behaviors. Evidence supporting the concept that altruistic potential is a common feature in human populations is developed. To go into greater depth in assessing critical features of the social brain, the two extremes of selfish–selfless behavior, callous unemotional psychopaths and zealous altruists who take extreme measures to help others, are compared on behavioral traits, structural/functional neural features, and the relative contributions of genetic inheritance versus acquired cognitive learning to their mindsets. Evidence from population groups ranging from newborns, adopted children, incarcerated juveniles, twins and mindfulness meditators point to the important role of neuroplasticity and the dopaminergic reward systems in forming and reforming neural circuitry in response to personal experience and cultural influences in determining behavior in the selfish–selfless spectrum. The underlying neural circuitry differs between psychopaths and altruists with emotional processing being profoundly muted in psychopaths and significantly enhanced in altruists. But both groups are characterized by the reward system of the brain shaping behavior. Instead of rigid assignment of human nature as being “universally selfish” or “universally good,” both characterizations are partial truths based on the segments of the selfish–selfless spectrum being examined. In addition, individuals and populations can shift in the behavioral spectrum in response to cognitive therapy and social and cultural experience, and approaches such as mindfulness training for introspection and reward-activating compassion are entering the mainstream of clinical care for managing pain, depression, and stress.

## Introduction

In the mid-1800s, the French Philosopher Auguste Comte constructed the word altruism from the Latin *alteri* (“others”) to name his vision of a moral call to place the needs of others over one’s self-interests. Altruism has since been defined in many senses, including an extreme selflessness in undertaking actions benefiting others without evident self-benefit and incurring personal risk. The conundrum created by Comte’s concept continues to reverberate through social debate, philosophy, theology, and biology, highlighting complex issues in the spectrum of behavior ranging from extreme selfishness to extreme selflessness (Ricard, 2015). The very concept of altruism raises important issues underlying two sharply divergent views of natural social order.

Philosophical, political and biological arguments on whether humans are naturally selfish or unselfish have flared for centuries and continue today. Thomas Hobbes contending in his work *Leviathan* printed in 1651 supporting strong Monarchist governments and running through current culture in Ayn Rand’s popular works assert there is a natural “universal selfishness” manifest in humans, with all behaviors characterized as altruistic being in reality actions that in some measure were in the actor’s best interest. Rand’s continuing influence on political discourse can be seen in the powerful American Speaker of the House and former Vice Presidential candidate Paul Ryan’s attribution of Rand’s *Atlas Shrugged* as formative in developing his political principles (Weiner, 2012). In Biology, the Oxford University Lecturer and popular science author Richard Dawkins has proclaimed, “We are survival machines – robot vehicles blindly programmed to preserve the selfish molecules known as genes” (Dawkins, 1976).

Sharply alternate opinions more supportive of Comte’s vision have also resonated for centuries and continue unabated. Two highly influential 18th century philosophers David Hume and Jean Jacques Rousseau argued that by nature humankind is unselfish. A contention strongly supported today by the prolific neuroscientist and popular science author Richard Davidson (Davidson, 2015). A middle position emphasizing a dual nature for humankind was presented in the 15th century essay by Pico della Mirandola *Oration on the Dignity of Man*, asserting that we can shape our own destiny by freely choosing whether to descend into brutish behavior or rise to the superior orders of the divine. This is a vision expanded upon by the Dalai Lama who wrote that “the most important thing in this existence of ours is to do something that can be of benefit to others. What we need more than anything is to develop an attitude of altruism – that is really what gives meaning to life” (Dalai Lama, 2007).

The second debate invariably accompanying any discourse on altruistic behavior is what is due to nature versus nurture. To better understand the scientific basis for addressing such profound social and philosophical issues, here we examine the biology and neurological basis of human altruism. We analyze the neural systems and the role of heredity, both genetic and neuron-based (cultural and social), in the development of behavior in the selfish–selfless spectrum, with the goal of discovering how and why portions of the population experience dramatically differing levels of empathy and compassion that strongly influence their worldview and role in society.

## Evolution of the Prosocial Brain

The term “altruism” has meant many different things in different times and places. Since Comte’s moral call to place the needs of others above one’s own needs, different disciplines have applied different definitions, and the semantics are themselves a necessary starting point (West et al., 2007). Group selection theory explains behavior such as kin sacrifice in terms of gene survival as opposed to individual survival (Simon et al., 2013; Gardner, 2015). Therefore, according to this evolutionary theory, related individuals will be more likely to perform altruistic acts and decrease their own survival if it benefits the survival of a related individual that carries many of the same genes. This theory is supported by extensive evidence in the literature of preferential treatment of kin (Madsen et al., 2007), while others argue that group selection is an emergent property of natural selection by individual fitness (Zhang et al., 2014; Kennedy et al., 2018). One question is how kin-preference is identified and conferred by an organism. Kin-preference may be a function of the extensive time spent with and proximity to the relative as opposed to an ability to identify genetic relatedness, as argued by cases of cronyism and altruistic preference for close friendships (Stewart-Williams, 2008). From an evolutionary biology perspective, “altruism” or empathic acts could be selected for culturally as a sign of fitness (Taborsky et al., 2016), as attested to by examples of prosocial behavior for non-relatives across the animal kingdom (Field and Leadbeater, 2016; Wilkinson et al., 2016). A more semantically “true” form of altruism may have its roots in the parental instinct to care for offspring, and may explain why empathic behavior is more commonly observed in species with protracted periods of pre-adult growth (Preston, 2013) requiring extended rearing and the resultant passing of learned behaviors, called acquired cognitive learning, as well as “neuron-based heredity,” including social and cultural factors that may have genetic and cognitive elements (Gash and Deane, 2015), to come into play. Thus, the importance of passing to kin the learned behaviors promoting culturally selected traits of compassion may counterbalance the selective value of genes promoting extreme selfish behavior (Bell et al., 2009).

The concept of altruism as an enhanced parental instinct relies on the evolution of several factors in both the altruist and the recipient: signaling of kinship status and need for compassion, recognition by kin of the signals, and donation-behavior by the kin (Sinervo et al., 2006). While this behavioral signaling mechanism may underlie parental instinct and compassion which is probabilistically directed toward kin, it is possible that simple parental behaviors – such as offspring retrieval, sustenance and shelter sharing, and emotional comforting – are behavior patterns of signaling-recognition-action that have been enhanced by evolutionary mechanisms (Preston, 2013) resulting in broader altruistic behavior from prosocial brains with greater capacity for receiving and passing on experience and acquired information. And as recent studies have shown, parenting-associated prosocial helping behaviors not only enhance the survival of the offspring, but also promotes better health, slower decline in functioning levels and lower risk of mortality for care-givers (Brown and Brown, 2015). Collectively, the evidence indicating prosocial altruistic capability provides for complex interactions that have come to form the foundation of our civil, societal interactions (Matusall, 2013). Social interactions often extend not only to members of our families, but to other members of our own social species, and often to members of other domesticated species on which we depend for our survival and social well-being.

## The Selfish–Selfless Spectrum

**FIGURE 1:**
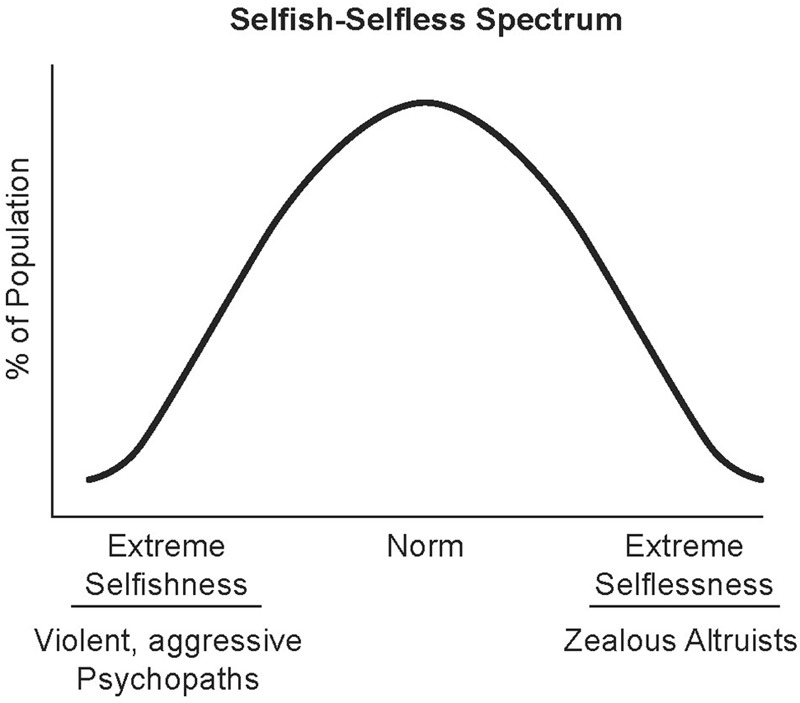
Selfish–selfless spectrum. The spectrum of human behavior from extreme selfishness to extreme selflessness is plotted here as an inverted U-Shaped curve. Social and cultural factors influencing perceptions are posited to shift the curve for individuals and populations to the left (e.g., racial hatred) or the right (e.g., compassion training). Illustrated by Matt Hazard.

Human evolution, especially since the separation from the last common ancestor shared with the great apes, is posited to have been driven by bipartite hereditary processes involving genetic and neuron-based systems (social and cultural heredity) (Gash and Deane, 2015). The development of large interactive social groups that share resources and work cooperatively toward accomplishing common goals distinguishes humans from the other great apes. The survival and success of large cooperative societies requires most of their members to mute their innate selfish drives and strengthen their selfless behavior. Converging evidence that will be reviewed here strongly supports that complex combinations of genetic and neuronal factors, including parenting, underlie the spectrum of selfish–selfless behaviors. Given the gaps in knowledge in this multidisciplinary area of research, we propose the spectrum be initially plotted as an inverted U-shaped curve with the *x*-axis representing the range from extreme selfishness to extreme selflessness and the *y*-axis representing the percent population at each point (**Figure 1**). We also propose that the extreme selfishness end of the spectrum is exemplified by callous-uncaring psychopaths and the extreme selflessness end by zealous altruists that take extreme measures to help others. We hypothesize that the landscape and peak of the curve shifts for given populations based on social and cultural factors (neuronal-based heredity) and genetic makeup.

## Cultural and Biological Influences on the Selfish–Selfless Spectrum

Differences in degrees of altruistic and prosocial behavior have long been noted between cultures. In one classic study published in 1975 that observed children’s behavior across six different cultures, it was found that 100% of children between the ages of 3 and 10 exhibited altruistic or prosocial behavior in Kenya, contrasted with only 8% in the United States (Whiting et al., 1975). Furthermore, this difference was linked to a cultural difference between the groups, especially in family function. Prosocial children were correlated with families where the women contributed economically and where the children were assigned tasks within the home. Supporting these observations is a study by Eisenberg and Mussen (1989) that found that Mexican children, Hopi children, and Israeli children were more prosocial than middle-class American children (Eisenberg and Mussen, 1989). Finally, Robarcheck and Robarcheck (1992) compared two cultures in environmentally similar conditions but with drastically different cultures, the cooperative Semai in the Malaysian rainforest and the individualistic and war-like Waorani from the Amazon (Robarcheck and Robarcheck, 1992). The Semai people exhibit prosocial and altruistic behavior, whereas the Waorani behave selfishly and reportedly save themselves if faced with danger as opposed to helping members of their society or family. These and other studies implied that societies that tend to focus on individual achievement and “success” result in children that are less prosocial and that exhibit fewer altruistic tendencies. Using the Price equation, researchers mathematically estimated that culture (neuron-based heredity) has more than one order of magnitude greater influence than genes on altruism and prosocial behavior at the population level (Bell et al., 2009).

Some studies have attempted to identify how the depth of social interactions an individual has is reflected in the gross anatomical structure of the human brain. For instance, a positive correlation has been recorded between the number of individuals a person regularly interacts with and the size of their amygdala bilaterally (adjusted for total intracranial volume), but not the hippocampus. This correlation also held true for the number of different social groups to which a person belongs, not just the number of friends with whom an individual interacts (Bickart et al., 2011). The amygdala is responsible for many automatic processes that influence social cognition (Adolphs, 2009) ranging from the more mundane such as fear, vigilance (Whalen, 1999) and alertness (Whalen, 2007) to the parsing and evaluation of facial features (Adolphs et al., 1994; Winston et al., 2002; Adolphs et al., 2005; Schiller et al., 2009) in conjunction with the fusiform cortex (Hadjikhani and de Gelder, 2003; Britton et al., 2008). The amygdala may play a central role in regulating an individual’s aversion to or propensity for social interaction based on the activation of the brain’s reward mechanisms during the reading of facial expressions and the subsequent regulation of comfort in social situations.

An interesting sub-population is political orientation, which is increasingly recognized as having a genetic as well as a social and cultural influence (Kandler et al., 2012; Hatemi and McDermott, 2012), including dopamine neurochemical receptor variant expression (Settle et al., 2010). In these sub-populations we again see the role of the amygdala in making social-based snap judgments when presented with images of faces. Bilateral amygdalar activation recorded by fMRI positively predicts a participant’s snap decision to vote for a person based solely on appearance, a phenomenon that was observed across cultures (Rule et al., 2010). Schreiber et al. (2013) found increasing brain activity in the right amygdala amongst Republican voters versus increased anterior insular activation in Democrats, suggesting different limbic processes are involved in reaching decisions in a risk-taking task. These findings were supported in separate studies by Kanai et al. (2011) reported that the gray matter volume of the right amygdala was observed to be larger in individuals self-described as more conservative, contrasted with those self-described as more liberal who exhibited greater gray matter volume in the anterior cingulate cortex (ACC).

The ACC is involved in many executive level brain functions, including reward-based decision making, error detection, and conflict monitoring. An example of a task that activates the error detection/conflict monitoring function of the ACC is the “Stroop task” (Pardo et al., 1990). In this task the name of a color is written in a different color of ink, for example the word “RED” is written in blue ink, and the subject is asked to name the word and ignore the color of the ink (or vice versa). This task activates the ACC. The ACC also may serve as an evaluative role after effortful error commission, producing emotional distress associated with the act of producing an error (Bush et al., 2000). Thus, the ACC is thought to be responsible for adapting behavior in response to the production of errors (Luu and Pederson, 2004). The perigenual region of the ACC may also play a role in modulating the reward mechanisms in a way perceived as gratitude at the relief of a stressor (Fox et al., 2015) and as the result of positive social interactions (Van den Bos et al., 2007). As a part of the social species, these functions may be critical in maintaining alliances and raising offspring through a protracted stage of dependency.

In one study (Christoy-Moore, 2016), subjects’ donations to individuals based solely on profiles listing socio-economic status were recorded (called the Dictator Game) and then activity levels in the subjects’ brains were measured by functional magnetic-resonance imaging (fMRI) while observing video of a human hand being punctured by a hypodermic needle, touched by a cotton swab, or static without stimulation (called the Needle Test). Subjects’ offers to low socio-economic status players in the Dictator Game were positively correlated with the subject’s own Empathic Concern score determined by questionnaire. A correlation was also observed between offers and blood-oxygen level dependent increases in fMRI activation of the primary somatosensory cortex and other associated areas during the observation of the painful hand stimulation, supporting a hypothesis of empathic behavior as a form of “self-other resonance” as a result of “neural resonance” between individuals. While undergoing fMRI the subjects were then asked to imitate faces that were displayed to them. The subjects who donated more money to low socio-economic players tended to exhibit greater levels of BOLD increases in fMRI in the left amygdala and also the left fusiform cortex which is a region responsible for facial processing and implicated in empathy.

Kim et al. (2010) observed a genetic variation on the oxytocin receptor gene OXTR at rs53576 between cultures (Kim et al., 2010). In individualistic European Americans a guanine (G) is more prevalent at this position, but in collectivist East Asians an adenine (A) is more common. Oxytocin is a neurohormone that is primarily involved in stimulating contractions of the uterine wall during childbirth and the milk “let-down” reflex of lactation during nursing. Oxytocin also acts on the central nervous system for brain development and to regulate behavior including maternal behaviors such as infant response and protection, and other social behaviors including bonding, trusting, and encouraging generosity (Yang et al., 2013). Nasally administered oxytocin reduces fear and anxiety (Kirsch et al., 2005), increases trust (Kosfeld et al., 2005), reduces xenophobic outgroup rejection (Marsh et al., 2017), increases monogamous behavior (Scheele et al., 2012), increases empathy (Sheng et al., 2013) and conformity with in-group members (Stallen et al., 2012). Some benefits of oxytocin beyond promoting positive social interactions include anti-inflammatory effects and indications for quicker wound healing (Gouin et al., 2010). It is being investigated as a treatment for the social deficits of autism (Dadds et al., 2014a), however, the efficacy reported in initial studies has been mixed with results ranging from modest benefits to no observed improvements (Young and Barrett, 2015).

Vasopressin has also been identified as a possible regulator of compassionate behavior. Human studies of individuals that exhibit strong sibling bonding (Bachner-Melman et al., 2005) and pair bonding (Walum et al., 2008) have identified that increases in the length of vasopressin 1a receptor repeat sequences 1 and 3 are linked to these behaviors in these sub-groups. Vasopressin, also called antidiuretic hormone or arginine vasopressin hormone (AVP), is a neurohormone that controls the antidiuretic effect through water reabsorption in the kidneys’ nephrons and by controlling the constriction of blood vessels. In terms of social behavior, central vasopressin receptors AVPR1a in the ventral pallidum of the prairie vole are necessary for pair bonding and partner selection (Lim and Young, 2004) by activating the reward circuitry during mating (Pitkow et al., 2001). This and other central AVP receptors have been shown to play critical roles in social recognition and interpretation of social cues as well as related stress pathways in knockout mouse models (Bielsky et al., 2004; Wersinger et al., 2004).

In addition to the strong evidence in animal studies that oxytocin and vasopressin are two neurohormones which play important roles in social behavior and the resultant reward and stress pathways that support those behaviors, there is increasing research supporting the hypothesis that genetic variants of OXTR and AVPR1a are predictive of humans displaying greater degrees of altruistic, empathic and compassionate behavior traits within population sub-groups. For example, Poulin et al. (2012) have reported that the amount of individual involvement in charitable activity and civic duties correlated with genetic variants. As expected, it was found that those with the OXTR rs53576 G to A variation or AVPR1a RS1/RS3 long to short variation were more likely to exhibit “prosocial” behavior by being more trusting of strangers, contributing more to charitable activities and participating in more civic duties.

## Extreme Selfishness: Criminal Psychopathy

Callous-unemotional criminal psychopaths epitomize extreme antisocial behavior. These individuals are characterized by aggression and violence with a long criminal record and frequent incarceration. Their core behavioral pattern of pervasively violating the rights of others without remorse can begin as early as 3 years of age and continue into adulthood (Hare, 2006; Gao and Raine, 2010; APA, 2013). In the United States, they are estimated to represent 16% of male prisoners (Kiehl and Hoffman, 2011). In England and Wales, the estimates are lower, close to 8% of men and 2% of women (Coid et al., 2009b), perhaps due to cultural differences between the countries. Serial killers fall into this category. However, by maintaining an outwardly normal persona, they can often evade detection and arrest for periods running into decades.

The spectrum of personality disorders classified as psychopathic is much broader than those on the extremist criminal end. Psychopaths can be separated into two groups – unsuccessful and successful (Gao and Raine, 2010). The unsuccessful are the callous-uncaring criminals. Successful psychopaths are a more diverse group ranging from ruthless con artists to leading statesmen (Dutton, 2016). Both unsuccessful and successful psychopaths can exhibit varying combinations of traits, which collectively predict their behavioral patterns. With the legal and societal problems created by criminal psychopaths, most research has been focused on defining their psychological features and neurobiology. The current criteria for determining if someone is a criminal psychopath is the Psychopathy Checklist-Revised (Hare, 2003; Babiak and Hare, 2006), which is crafted for clinical and legal use, emphasizing antisocial and criminal behaviors. It is used worldwide and its influence is seen in the gold standard for clinical diagnosis, the 5th Edition of the American Psychiatric Association’s *Diagnostic and Statistical Manual of Mental Disorders* (APA, 2013) where psychopathy is described as a synonym for Antisocial Personality Disorder (see **Table 1**).

**Table 1:** PCI-R: psychopathy criminal focus (APA, 2013).

• Core features: Callous-unemotional – lacking empathy and lacking remorse for mistreating others.
• Frequently breaking the law, being arrested.
• Pervasive dishonesty, chronic lying.
• Dysfunctional planning.
• Impulsive, irritable and aggressive behavior.
• Fearless, reckless and irresponsible, endangering self and others.

As Hare developed his PCL-R as a research tool based largely on his experience in analyzing criminals, its use more broadly in formulating public policy, in business and in conducting unbiased social research is controversial (Skeem et al., 2011). Also, it has raised a major scientific issue. Is psychopathy a monolithic disorder (qualitative), or is it a syndrome with multiple interacting factors determining the extent and phenotypic expression (quantitative)? To more fully evaluate the hypothesis of multiple interacting factors, an alternate rating scale to the PCL-R has been developed, the Psychopathic Personality Inventory-Revised (PPI-R, see **Table 2**) designed and validated to measure more affective and interpersonal traits and to be used in both criminal and non-criminal populations without *a priori* assumptions of antisocial and criminal behavior (Skeem et al., 2011; Dutton, 2016; Sorman et al., 2016).

**Table 2:** PPI-R: psychopathy positive and negative features (Skeem et al., 2011; Dutton, 2016).

• Coldblooded • Powerful social dominance • Fear immunity • Stress immunity • Machiavellian self-interest • Rebellious non-conformity • Blame externalization • Carefree living (no planning)

While the two different rating scales overlap in measures such as meanness (e.g., callous and unemotional, coldhearted), antisocial behavior (e.g., pervasive dishonesty, Machiavellian self-interest) and poor planning skills, PPI-R includes the positive traits of boldness (social dominance, immunity to stress and fear).

While all unsuccessful psychopaths are by definition criminals, as mentioned before, successful psychopaths on the PPI-R scale are found in politics, medicine and business. Their actions range from criminal to courageous, as evidenced by the high PPI-R scores of national leaders of World War II: Franklin D. Roosevelt, Winston Churchill, and Adolph Hitler (Dutton, 2016). Roosevelt and Churchill were powerful leaders whose bold, hardnosed (coldblooded) actions were instrumental in the survival and success of their social order; Hitler’s leadership epitomizing callousness, lack of remorse and blame externalization was disastrous for all of Europe and led to notorious crimes against humanity.

As successful psychopaths can intelligently conceal their psychopathic traits, their number in the population is difficult to detect. In one large population survey, the prevalence of successful (i.e., no criminal record) psychopathic individuals living in households in England was 0.6% (Coid et al., 2009a). In the professional world of politicians, businessmen, doctors and lawyers, the number may be much higher. In their book *Snakes in Suits: When Psychopaths go to Work*, industrial psychologist Babiak and criminal psychologist Hare (Babiak and Hare, 2006) estimated 3.5% of professionals in business possess strong psychopathic traits. While some professionals with psychopathic traits are criminals, others benefit the social order by boldly leading changes needed for cultures to adapt to ever changing environmental, economic, and political conditions.

## Neurobiology of Psychopathy

Dysfunctional emotional processing is a defining feature of psychopathy (Anderson et al., 2017), from lacking empathy to possessing immunity to stress and fear. Meta-analyses of 26 studies found emotional recognition of facial expressions and vocal cues was significantly impaired in young and adult psychopaths for all of the basic emotions: anger, disgust, fear, happiness, sadness, and surprise (Dawel et al., 2012). Their inability to recognize fear and sadness was especially pronounced, but they also exhibit reduced neural response to laughter (O’Nions et al., 2017). Such blunted emotions affect perceptions, thought processes and actions toward others, fostering both boldness and lack of remorse. Dysfunctional emotions also affect another trait of criminal psychopaths – deeply flawed reasoning, including moral judgment.

**FIGURE 2:**
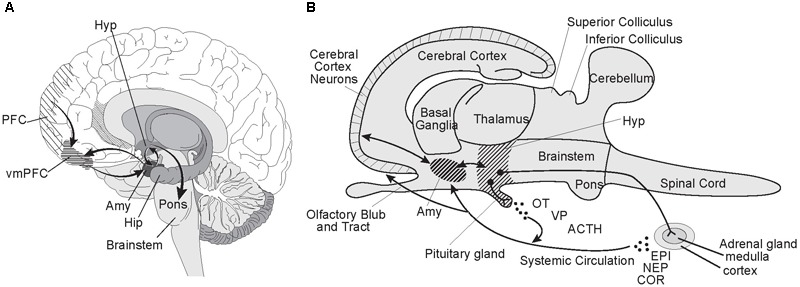
Prefrontal cortex-amygdala-hypothalamic circuitry. The prefrontal cortex-amygdala-hypothalamus axis has a pivotal role in social-emotional processing. Developmental disorders and injuries effecting its neural assemblages and circuitry can lead to antisocial behaviors characterizing psychopathy. **(A)** In this parasagittal view of the human brain, the spatial relationships and neural connectivity between the prefrontal cortex (PFC), amygdala (Amy), and hypothalamus (Hyp) are illustrated. Lesions involving the ventromedial PFC (vmPFC) are especially disruptive of prosocial behavior. Note the central role of the amygdala in the circuitry linking the prefrontal cortex and hypothalamus in the emotional processing network. Also see its intimate integration of the amygdala with the head of the hippocampus (Hip), which initiates and consolidates cognitive memory and learning processes in the brain. **(B)** Here is a schematic based on the generic mammalian brain to illustrate the multiple actions taken by the hypothalamus when a “Fight or Flight” response is triggered by cortical-amygdala interactions signaling high levels of risk or immediate danger, including life or death situations. Neurons in the hypothalamus terminating in the pituitary release oxytocin (OT), vasopressin (VP) and adrenocorticotropic hormone (ACTH) into the systemic circulation. The sympathetic nervous system is fully activated via the hypothalamus, including by a direct neural projection to the adrenal medulla stimulating release of epinephrine (EPI, adrenalin) and norepinephrine (NEP, noradrenalin) into the blood stream. ACTH stimulates the release of the stress hormone cortisol (COR) from the adrenal cortex. The physiological responses include hyperarousal, focused vision, increased heart rate and blood pressure, blood shunted to the muscles, and suppression of digestion and appetite. Illustrated by Matt Hazard.

Three of the sites in the brain responsible for criminal psychopathic behavior are also principal components of the neural circuitry for normal social-emotional processing: the *prefrontal cortex* (PFC), *amygdala*, and *hypothalamus* (**Figure 2**). Psychopathic behavior resulting from injury or disease implicates the ventromedial PFC (vmPFC) as a critical node for prosocial behavior; its dysfunction resulting in antisocial behavior (Anderson et al., 1999; Barrash et al., 2000; Trebuchon et al., 2013). The enlarged *prefrontal cortex* is the neocortical (i.e., evolutionarily newest) region of the human brain responsible for top down, executive control; while the amygdala is the part of the allocortical (i.e., evolutionarily old) cortex that, as discussed earlier, integrates sensory and acquired information, including facial features, to assess threat levels. In the mammalian brain, the PFC is richly networked with the *amygdala* (McDonald et al., 1999), with neurons in the cerebral neocortex sending fibers to connect with neurons in the *amygdala* embedded in the rostral temporal lobe (Stein et al., 2007). Like most neural assemblages in the brain, it is a two-way street with *amygdaloidal* neurons sending fibers to the cerebral cortex. The *amygdala* in turn is richly interconnected with the *hypothalamus* (Herman, 2012), another evolutionarily ancient brain region that regulates homeostasis and autonomic nervous system activity (Jansen et al., 1995) and controls neuroendocrine functions including secretion of oxytocin and vasopressin into the systemic circulation (Carter, 2014).

The association between medial *prefrontal cortex* (PFC) dysfunction and psychopathic behavioral features including lack of empathy and remorse, dishonesty, and poor planning and decision-making skills, has been extensively documented since the index case of Phineas T. Gage in 1848 (Harlow, 1868; Damasio et al., 1994). Since then, there has been an abundance of research supporting the role of the PFC in social processing and behavior regulation (Anderson et al., 1999; Grossman, 2013). In individuals with pronounced conduct control problems, numerous studies have shown the *amygdala* is smaller along with less gray matter volume in the frontal and temporal cortices (Yang et al., 2010; Rogers and De Brito, 2016). Hypoactive *amygdala* responses to stimuli of others in distress are characteristic of children with the callous-unemotional trait and associated with aggressive behavior (Lozier et al., 2014). Humans with bilateral *amygdala* lesions have impaired learning of fear and responding to eminent danger (Bach et al., 2015; Klumpers et al., 2015). Bilateral *amygdala* lesions in rhesus monkeys have significantly blunted stress responses (Raper et al., 2013). These findings strongly link the behavioral traits of the callous-unemotional trait, boldness, and fear/stress immunity to *amygdala* functions.

The *hypothalamus* is intimately involved as the control center of the brain for autonomic responses and regulation of sex hormones and the secretion of oxytocin, cortisol, and vasopressin into the bloodstream. Low oxytocin levels have been linked with callous-unemotional scores in adolescents (Levy et al., 2017). Supporting this link are other studies indicating that inactivation of the oxytocin receptor by DNA methylation is correlated with an increased risk of callous-unemotional traits (Cecil et al., 2014). Children and adolescents with the callous-unemotional trait exhibit reduced cortisol response (von Polier et al., 2013; Grotzinger et al., 2018) perhaps explaining increased boldness/impulsivity, and are at high risk for developing into criminal psychopaths (Kahn et al., 2013; Kimonis et al., 2016). As discussed earlier, oxytocin and vasopressin are two neurohormones that have essential roles in social behavior and the resultant reward and stress pathways that support those behaviors (Poulin et al., 2012).

**FIGURE 3:**
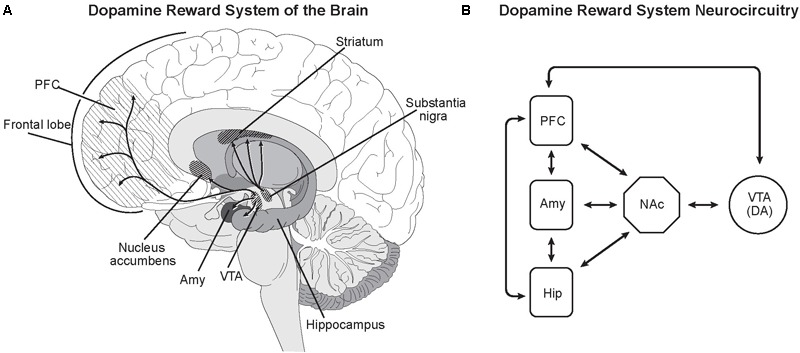
Dopamine reward system of the brain. **(A)** Dopamine neurons in the ventral tegmental area (VTA) directly innervate the nucleus accumbens (NAc), prefrontal cortex (PFC), amygdala (Amy), and hippocampus (Hip) (de la Mora et al., 2010; Kahn and Shohamy, 2013; Shnitko and Robinson, 2014). As in most neural networks, connectivity between the nuclei goes both ways. Dopamine release from the VTA promotes feelings of satisfaction, pleasure and euphoria, rewarding and motivating behavior. Dopamine release from the substantia nigra in the striatum modulates motor functions. **(B)** While the neurocircuitry modulating the VTA and NAc is complex, major projections from the PFC, Amy and Hip to the VTA and NAc have been identified (Sesack and Grace, 2010). This is consistent with known modulation of reward system dopaminergic activity being influenced by goal-directed behavior (PFC), emotions and feelings (Amy) and experience/memories (Hip) (Sesack and Grace, 2010). Illustrated by Matt Hazard.

The neural circuitry for the Impulsive-Antisocial dimension of psychopathy overlaps with that for Boldness, but differs in important details. The size of the *striatum*, a major component of the basal ganglia, is larger in Impulsive-Antisocial individuals (Korponay et al., 2017), especially the *putamen* and the *nucleus accumbens* in the *ventral striatum*, the reward center of the brain (**Figure 3**).

Dopamine release from *ventral tegmental area* dopamine neurons innervating the *nucleus accumbens* is an essential component of the reward circuitry (Sesack and Grace, 2010; Kahn and Shohamy, 2013). In functional MRI studies, the *ventral striatum* including the *nucleus accumbens* displays robust activation in criminal psychopaths in game tasks involving rewards (Pujara et al., 2014). The *nucleus accumbens* is a major component of the limbic system (Morgane et al., 2005), the part of the human brain that provides emotional processing and motivational information to the enlarged, more deeply layered areas of the prefrontal cerebral cortex (Shnitko and Robinson, 2014). Other interacting centers include the *amygdala* and *hippocampus* (de la Mora et al., 2010).

As recently emphasized by Reidy et al. (2017), reward-dominant learning and decision making in callous-unemotional individuals with strong Impulsive-Antisocial traits can explain their extremely violent behavior, their hair-trigger response for evoking rage or anger (the fight component of “Fight or Flight” behavior). In these individuals, rage and impulsivity with reduced fear from the *amygdala* evokes pleasure – dopamine release in the *nucleus accumbens*.

The absence of normal fear and stress responses is analogous to removing the brakes on a bulldozer. Boldness and greatly muted fear with positive reinforcement from the reward system of the brain is the result. For criminal psychopaths, it leads to clashing violently with others and taking risks that lead to incarceration, debilitating injuries or death.

## Nature Versus Nurture in Psychopathy

Based on parent reported data on 5092 twins, genetically modeled inheritance for the core feature of psychopathy – callousness and unemotional trait – was 70% (Henry et al., 2016). A smaller more recent study examining the reliability of parent reported data refines the estimate to 47% (Moore et al., 2017). For the traits of Boldness and Impulsive-Antisociality, twin studies have reported inheritance in the 40–50% range (Blonigen et al., 2005) and indicating the two traits were not linked genetically and differ in their neurobiology.

Some progress has been made in identifying genes associated with features of psychopathy. Consistent with data reported throughout this review that oxytocin has a prominent role in promoting prosocial human behavior, two independent studies have recently found a high association between an oxytocin receptor gene and callous-unemotional traits (Beitchman et al., 2012; Dadds et al., 2014b). Endogenous oxytocin is important in neural development including neural circuitry (Carter, 2014), making it difficult to predict the effects from administration of exogenous oxytocin to the adult brain after critical periods in brain development. Initial studies indicate nasal administration of oxytocin increases aggressive behavior in normal adults (Ne’eman et al., 2016) and adults with antisocial personality disorder (Alcorn et al., 2015).

### Role of Neuron-Based Heredity in Psychopathy

To what extent can neuronal-based heredity – learned societal and cultural traits – compensate for strong genetic psychopathic predispositions? This acquisition of social and cultural information begins before birth (Partanen et al., 2013) as newborns recognize their mother’s voice and can distinguish it from a stranger’s voice (Beauchemin et al., 2011). Prenatal development and functioning of neural systems is evident after birth with the pattern of crying of newborns shaped by their native language (Mampe et al., 2009). Positive parental support and maternal behavior is exceptionally important during these critical periods. Chaotic home environments, negative parental behavior and mothers with strong callous-unemotional traits can affect their fetus’s and infant’s emotional and cognitive development (Fontaine et al., 2011; Hyde et al., 2016; Viding and Pingault, 2016).

In a study of 561 children adopted within several days after birth where severe callous-unemotional maternal behavior was replaced by strong positive support, major effects were found in altering behavior (Hyde et al., 2016). Having a biological mother with severe callous-unemotional behavior predicted the same traits in their children at 27 months of age, even though they had not been parenting them after adoption. Positive reinforcement by the adoptive mother significantly mitigated the expression of callous-unemotional behavior in their adopted child. The effect was dose-dependent, adoptive mothers with high positive reinforcement completely buffered the expression of the callous-unemotional behavior at 27 months (Hyde et al., 2016; Viding and Pingault, 2016). Follow-up studies over time will be extremely important to assess the efficacy of early positive parenting on adolescent and adult behavior patterns.

Even with the caveat that longer longitudinal studies are needed, the malleability of callous-unemotional behavior in early childhood is encouraging. But as a developmental neural disorder with structural neuroanatomical abnormalities (Cope et al., 2014) and impaired functional connectivity (Harenski et al., 2018), how long is the window of opportunity open for therapeutic intervention for criminal psychopaths? Working with juvenile delinquents, over half of whom had committed a serious violent felony, a program at the Mendota Juvenile Treatment Center in Wisconsin using intensive therapy balancing punishment for bad behavior with rewards for improved behavior was able to reduce the number of crimes by over 35% perpetrated by the trainees over a 4–5 years period after release, compared to a treatment-as-usual control group (Caldwell and Van Rybroek, 2005; Caldwell, 2011). While the effects on violent behavior were impressive, there remained a significant risk for aggressive behavior injuring others.

The Mendota Center approach of effectively reinforcing the reward center of the brain for improved behavior, while providing negative reinforcement for bad behavior, is now being tested elsewhere for younger adolescents with strong callous-unemotional traits (Hagerty, 2017). However, treatments for adult criminal psychopaths have been notoriously ineffective and upon release 90% commit another violent crime within 20 years (Anderson and Kiehl, 2014).

## Extreme Selflessness: Zealous Altruism

Placing the interests of others above one’s own safety occurs so regularly in the United States and the rest of the world that it garners little media attention (Ricard, 2013, 2015). Only when there is serious injury or death of the Good Samaritans does it make the news, such as in May 2017 when a crazed man approached two young women on a commuter train in Portland, Oregon brandishing a knife and screaming anti-Muslim insults. Three strangers rushed to rescue the girls. Two died and the third was seriously injured (Kristof, 2017). As the list of Carnegie Medal recipients for *Extraordinary Civilian Heroism* shows (CarnegieHero.Org, 2017), altruists range from adolescents to aged adults. Some are very young like 10 year-old Kiera Larsen who saved a 2-year old child from being run over by a car that then struck and killed her. They can be old, like 72 year-old Louis Scharold who braved intense heat from burning, wrecked trucks to reach through the broken windshield of one vehicle to pull the dazed driver to safety. Twelve of the 94 Carnegie Medal Recipients in 2016 lost their lives in trying to save others. Again, routine altruistic actions are commonplace and seldom make the news, but extraordinary risks taken by some can and do lead to injury and death. These zealous altruists on the extreme end of the spectrum are those who take extreme measures to help others, unnecessarily placing themselves in harm’s way, such as anonymous living kidney donors that partake in surgery to donate an organ to an unrelated and unknown recipient they will never meet (Tong et al., 2012).

Altruism can be impulsive suggesting instinctive reactions as in the preceding cases, or premeditated by choosing to help others in ways that are knowingly risky indicating involvement of executive functions, such as the actions of David Eubank, the American Aid Worker who rescued a young girl, the lone survivor of about 70 civilians massacred by ISIS fighters as they tried to free Mosul in June of 2017. Braving sniper fire with some support from Iraqi and US Forces, Eubank ran into the street, picked the girl up and brought her back to safety (Yuccas, 2017). Eubank has repeatedly chosen to go to war-ravaged areas in Asia to aid children and others in need. Less dramatic, but equally extreme premeditated acts of generosity are those of altruists who donate one of their kidneys to help an unknown anonymous patient (Marsh et al., 2014), placing their own lives at risk from complications of elective surgery and the removal of an organ. Other less risky altruistic premeditated actions include being a blood donor or donating bone marrow for transplantation.

Thus, the conundrum of altruism – taking risks by placing the interests of others, often strangers, beyond one’s self-interest – seems to directly violate the “survival of the fittest” principle of gene-based evolution. Darwin noted both sides of the issue in *The Descent of Man*. He astutely recognized that selfishness was a roadblock to human social evolution, “Selfish and contentious people will not cohere, and without coherence nothing can be effected.” However, Darwin continued, writing, “He who was ready to sacrifice his life, as many a savage has been, rather than betray his comrades, would often leave no offspring to inherit his noble nature” (Darwin, 1871). Darwin (1871) proposed that as ancestral human reasoning and foresight powers increased, the benefits of reciprocal social assistance would become obvious and gradually lead to inherited reciprocal benevolence.

Darwin seems to have been justified, as just discussed there is strong evidence for both instinctive and cognitive benevolence in our species. This evolution has occurred thanks to a capability of the mind Darwin did not anticipate. Namely, our species possesses enhanced mind-reading skills to understand our own thoughts and emotions and what others are thinking and feeling (Heyes and Frith, 2014; de Waal and Preston, 2017). Affective perception of other’s emotions includes the six basic emotional states visualized by facial expressions and body language: anger, fear, surprise, sadness, disgust, and happiness. Feeling and understanding the emotions of others, the recognition of signals of need, sets the stage for perception-based actions such as benevolence and compassion.

With mind-reading skills at work, two major interactive factors appear to be crucial for promoting and maintaining broad levels of cooperation within human populations. The first, as hypothesized by Batson et al. (1981, 1983), Toi and Batson (1982) and tested in a series of studies, is a strong link between empathy and altruism. A linkage that has been repeatedly replicated (Persson and Kajonius, 2016) and further modeled and linked with oxytocin by Zak and Barraza (2013). The second factor is punishment of those violating social norms (Fowler, 2005). The efficacy of punishment as in promoting cooperation has been controversial. However, a meta-analysis of punishment and cooperation in 18 societies found punishment strongly promoted cooperation in societies with high trust levels (Balliet and Van Lange, 2013). Also as Mussweiler and Ockenfels (2013) have demonstrated, perceived similarity promotes altruistic cooperation as well as evoking increased punishment for norm violations. The result, they suggest, enhances the ability for similar individuals to build strong, stable socially cooperative groups.

## Neurobiology of Altruism

Implicit mind reading skills (gene-based) are present by the first 4–7 months of life with normally developing infants making face-to-face communication that activates the medial prefrontal cortex (Grossman et al., 2008; Kovacs et al., 2010; Urakawa et al., 2015). Most or all neurocognitive mind reading skills are acquired and strongly shaped by culture (neuron-based), with the early stages of learning similar to those of learning to read (Heyes and Frith, 2014). Infants are very vulnerable, so development of the ability to distinguish between those who can help versus those who may pose a risk is essential. Strong evidence from many research groups indicates that the majority of infants as early as 6–10 months of age prefer and selectively approach individuals displaying intentional prosocial behavior (Hamlin and Wynn, 2011; Holvoet et al., 2016; Van de Vondervoort and Hamlin, 2017). Also by 1–2 years of age, simple reciprocal interactions elicit an early form of altruistic behavior, the child helping the experimenter or a stranger obtain an object clearly out of their reach (Barragan and Dweck, 2014). By 5 years of age, children are capable of sharing with others and anticipating reciprocation (Sebastian-Enesco and Warneken, 2015).

**FIGURE 4:**
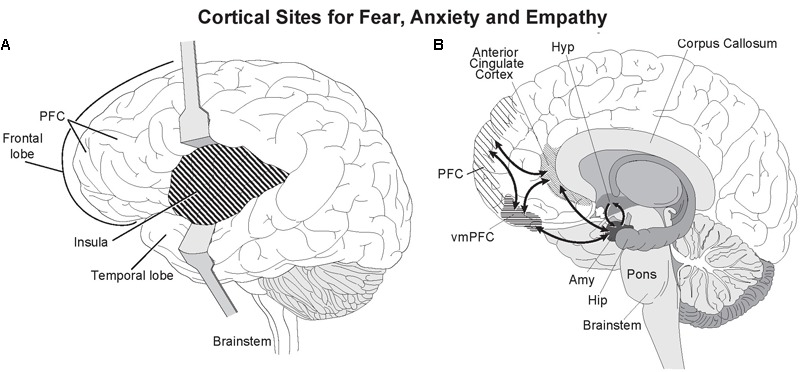
Active cortical sites for fear, distress, and empathy. Two deep cortical regions, the anterior insula and anterior cingulate cortex are strongly activated when feeling fear and empathy. Both are strongly interconnected with the amygdala (Stein et al., 2007). **(A)** The insula lies beneath the temporal and cortical lobes and can be seen by separating the two lobes. **(B)** The anterior cingulate gyrus is the deepest cortical region of the prefrontal cortex (PFC) as seen in this sagittal section, and caps the anterior corpus callosum. Illustrated by Matt Hazard.

Altruism goes well beyond reciprocation by compassionately helping a stranger with no apparent self-benefit and at some risk to one’s own being. Neuroimaging studies have provided important insights into the neural networks underlying the behavioral linkage between empathy and compassion. As defined by de Waal and Preston (2017), empathy is a “term for all processes that emerge from the fact that observers understand others’ states by activating their own personal, neural, and mental representations of that state.” Empathy then is a passive state of feeling. Physical pain or distress, and *empathy* from witnessing pain both activate the same higher brain areas, the *anterior insula* and anterior to mid *cingulate cortex* (**Figure 4**) (Lamm et al., 2007, 2011). Depending on the type of distress, other brain areas such as the primary motor and somatosensory cortices are recruited to simulate in the observer the neural activity in distressed individual: a vivid example of the continuing theater of the mind envisioned by the founder of modern psychology William James over 100 years ago where one’s own thoughts and sensations are blended with ongoing experience to produce emotions and feelings. *Empathy* for another in extreme pain can be extremely painful (Fitzgibbon et al., 2010; Ricard, 2015). Intense and repeated exposure to distress can lead to severe emotional and health problems including empathetic distress and post-traumatic stress syndrome (APA, 2013; Klimecki et al., 2014).

**FIGURE 5:**
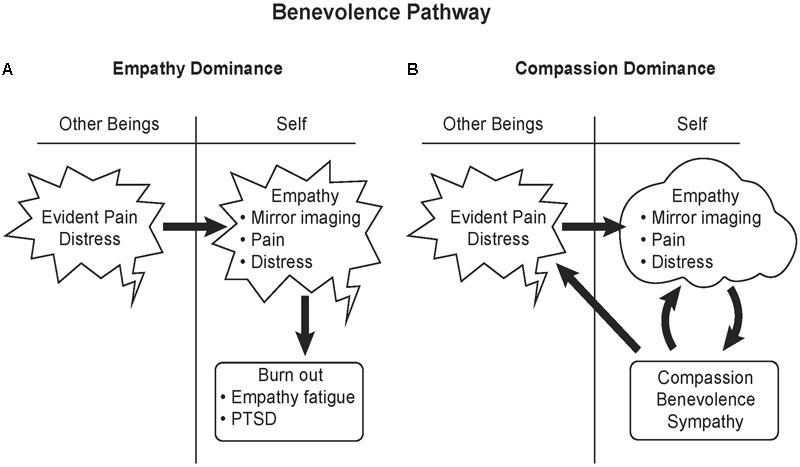
Benevolence pathway. **(A)** Empathy involves literally feeling another’s pain. An individual with empathic responsiveness upon seeing and or hearing others in pain mirrors that pain in their brain, activating the same higher cortical brain areas activated by fear, the anterior insula and mid-to-anterior cingulate cortex. Intense exposure to a stressful event or repeated exposure to stress in others can lead to burnout, empathy fatigue or PTSD. **(B)** Compassion activates the reward system of the brain and can significantly calm empathetic feelings of fear and pain. Therefore compassion and benevolence elicit positive feelings. Altruism not only benefits the recipient, but also benefits the altruist by rewarding their behavior with feelings of satisfaction. Illustrated by Matt Hazard.

As opposed to the passive state of empathy, compassion is taking action to help others, including other species, in distress. Synonyms for *compassion* that help define it are benevolence, kindness and sympathy. Compassionate actions activate the reward system of the brain, the ventral striatum and the dopaminergic ventral tegmental area, as well as the medial orbitofrontal cortex (Klimecki et al., 2014). The positive affect from compassion not only reinforces benevolent behavior, but can also calm painful empathetic feelings (**Figure 5**).

Neuroimaging studies on adult Europeans making decisions on altruistic giving have identified two strongly engaged brain areas. Activity in anterior insula predicted generosity in individuals influenced by emotional empathy, while activity in the temporoparietal cortical junction was associated with cognitive empathetic giving (Tusche et al., 2016). Another study taking a similar approach testing young European adults reported that volume of gray matter in the right temporoparietal cortical junction was strongly positively correlated with the maximal acceptable cost for an altruistic action (Morishima et al., 2012). The results from both studies are supportive of the concept of altruistic potential being a common feature in populations and that can be evoked by empathetic feelings.

Another approach for identifying brain areas engaged in altruistic giving is that taken by Marsh et al. (2014) using neuroimaging to quantify structural and functional differences between Zealous Altruists (individuals who had donated one of their kidneys for transplant recipients) and Criminal Psychopaths. Brain size can be larger than normal controls in zealous altruists and, even controlling for the brain volume, the right amygdala is larger (Marsh et al., 2014). As reviewed earlier, in psychopaths with conduct control dysfunction, the *amygdala* is smaller along with less gray matter volume in the frontal and temporal cortices (Yang et al., 2010; Pardini et al., 2014; Rogers and De Brito, 2016). In contrast to psychopathy where responses to all facial expressions of emotion are muted, altruists show an enhanced responsiveness to fearful facial expressions and diminished responsiveness to anger (Marsh et al., 2014).

The neural pathways involved in compassion are similar to those for impulsive-antisocial behavior. In this instance, prosocial compassionate behavior rather than antisocial behavior activates the dopamine reward system of the brain. The same neural systems are engaged (hippocampus, ventral striatum and ventral tegmental area, prefrontal cortex and amygdala). The major difference between prosocial and antisocial behavior is likely embedded in the perception of social order. This difference is wittily broached in a letter by Walpole (1769) in which he declared, “this world is a comedy to those that think; a tragedy to those that feel – a solution of why Democritus laughed and Heraclitus wept.” For altruists, the full capability for processing and integration of sensory and acquired information in the reading and feeling of emotions of others engenders taking positive actions – compassion – that mitigates the altruist’s own distress and reflexively activates the reward system. With the muted neurocircuitry for basic emotions in criminal psychopaths, taking action in the form of violent aggression actives the anger/fight component of the Fight or Flight response reflexively activating the reward system.

## Nature Versus Nurture in Altruism

Given that altruistic behavior can be expressed by large segments of the population as discussed earlier, the genes underlying altruism may be commonplace. This includes genes for central dopaminergic systems engaged in the reward system and genes for oxytocin receptors that promote development of the social brain with strong capabilities for communication and mind reading of emotions (Donaldson and Young, 2008; Carter, 2014; Heyes and Frith, 2014).

If genes underlying altruism are the norm, then it can be predicted that gene variants and mutations resulting in dysfunctional and antisocial behavior can be used to identify gene networks that are good candidates for promoting altruistic behavior. With the Callous-Unemotional trait found in individuals at high risk for violent criminal behavior, genetic variants in the oxytocin receptor have been identified as discussed earlier. Dysfunctional social behavior is also found with the overexpression of oxytocin. Williams Syndrome, which results from the loss of 28 genes, features dysregulation of oxytocin and vasopressin secretion by the hypothalamic-pituitary system (Dai et al., 2012). Baseline plasma levels of oxytocin are three-times higher and vasopressin levels 0.30% higher in Williams Syndrome than in controls. Individuals with Williams Syndrome possess cognitive deficits with behavior characterized by diminished social anxiety and fearfulness, readily approaching, socially interacting with and trusting strangers (Jarvinen et al., 2013). However, they experience difficulty interacting with peers and have high non-social anxiety. With cognitive deficits and overly trusting of others these individuals cannot live independently.

Several hundred genes, many of them rare copy variations, have been implicated as risk factors for dysfunctions in communication and mind reading that characterize autism spectrum disorders (Pinto et al., 2014; Sahin and Sur, 2015). The large number is consistent with the broad heterogeneity of symptoms and their expression in autism. While alterations in oxytocin stimulatory proteins, oxytocin plasma levels and genetic variance in oxytocin receptors have been reported as risk factors for autism (Jacobson et al., 2014; LoParo and Waldman, 2015), clinical trials on the efficacy of oxytocin therapy has found only modest benefits or no positive effects (Young and Barrett, 2015). Collectively, the genetics underlying psychopathy, Williams disease and autism indicate that no one gene is responsible for the development of the human prosocial brain, but a broad network of interacting genes found in normal populations set the stage for the second system of heredity (neuron-based) to sculpt prosocial brain functions.

### Role of Neuron-Based Heredity in Altruism

The human genome containing around 20,000 protein-encoding genes can provide the basic blueprint for brain development, but training and experiences in the early years from infancy through childhood are crucially important in sculpting brain development and function. As discussed earlier, prosocial behavior is displayed by most infants and preschool children. Culture plays a major role in enhancing and strengthening prosocial behavior. Even where genetic inheritance of the callous-unemotional trait is a significant risk factor for developing criminal psychopathic behavior, especially in chaotic, uncaring environments, expression of risky behavior can be muted by early adoption and raising with strong positive parenting.

The typical experience for a newborn is rapid bonding with a loving mother providing intensive high-attentive care. When this does not happen and there is profound social deprivation early in life, such as institutionalization in an orphanage, prosocial brain development is severely compromised. Connectivity between the prefrontal cortex and amygdala is altered and the amygdala-hypothalamic-pituitary stress axis affected (Gee et al., 2013; McLaughlin et al., 2014). The constellation of problems found in institutionalized children include: smaller brain size with reduced cortical thickness, deficits in cognitive and language functions, problems with emotional regulation, and increased risk for psychotic symptoms (Tottenham et al., 2010; McLaughlin et al., 2014; Trotta et al., 2015; Bick and Nelson, 2017).

From these studies we see that development plays an important role in cultivating behavior, and that early intervention can help recover normal development, even impacting adolescent criminals. How much of this is learning and how much is developmental determinism? Studies of economic games such as the Prisoner’s Dilemma indicate wide differences in the display of prosocial behavior in single and iterative play (Fehr and Schmidt, 2003). However, a study of 102 adults participating in a repetitive Social Gambling Task indicated that an individual’s ability to learn how their actions impacted another’s outcome led to more prosocial behavior (Kwak et al., 2014). In this context, learners were defined as those who made choices resulting in economic gain, whether for themselves or others, and is mathematical in nature. On a neuroscientific basis, learning occurs with the release of dopamine in the reward and reinforcement centers of the brain including regions of the striatum, such as the nucleus accumbens, from the ventral tegmental area and substantia nigra. Rodents have been observed to learn the conditions of an experimental shock more quickly after vicariously observing other rodents receive the shock during such conditioning experiments. These rodents vicariously learn the conditions of the experimental shock more rapidly if they also have experienced the shock for themselves, regardless of whether that shock was experienced within the context of the conditioning experiment or not (Sanders et al., 2013; Lahvis, 2017). Rodents that had these vicarious learning experiences while hearing pain-induced vocalizations in others exhibited increased activation of both dopamine and serotonin circuitry and the ACC (Kim et al., 2014) suggesting both empathy and learning through limbic systems and dopaminergic reward mechanisms. These studies on vicarious or empathic learning are supported by studies in humans showing experience of a painful stimulus increases empathy in human observers (Eklund et al., 2009; Preis and Kroener-Herwig, 2012) which coincide with BOLD fMRI data indicating that the perception of pain in others is neurologically similar to the actual sensation of pain (Preis et al., 2015). Participation in the Zurich Prosocial Game, a computer-based compassion training game that requires cooperation, has been shown to increase helping behavior in human subjects 5 days after training (Leiberg et al., 2011), suggesting long-term processing and learning through reward mechanisms. Reflecting on gratitude increases scores on a self-report measure of altruistic values and coincides with increased BOLD fMRI activation in the nucleus accumbens and vmPFC (Karns et al., 2017) indicating involvement of the mesolimbic dopamine pathway for reward and reinforcement learning of altruistic behavior (Strobel et al., 2011). Further supporting the role of social learning as important in the cultivation of prosocial behavior are studies linking oxytocin, previously described for its prosocial and parental functions, with dopamine circuitry. Oxytocin receptors and dopamine receptors coexist in the striatum, medial PFC, substantia nigra and ventral tegmental area (Skuse and Gallagher, 2009). Intranasal oxytocin in normal humans appeared to increase the reward for reciprocated cooperation through increased activation of the dopamine-activated, reward-linked nucleus accumbens during repeated iterations of Prisoner’s Dilemma game (Rilling et al., 2012) which supports the role of oxytocin and dopamine learning pathways in trust and reciprocation behavior.

Thus social and cultural behaviors and activities are mechanisms for not only training the brain beginning in prenatal life, they are also principal components in determining brain development and dopaminergic reinforcement learning in the adult. Empathy and compassion are feelings with basic genetic and neural underpinnings crucial for the development of large interacting social communities characterizing our species. Successful cultures have strong and effective implicit and explicit mechanisms for promoting and enhancing empathetic and compassionate behavior. Empathy and compassion are core values in most, if not all, of the world’s major religions. Buddhist methods for training the mind through meditation and yoga (Tomasino et al., 2014) are an example of positive approaches to enhance empathy and compassion that have been secularized and entered mainstream Western culture and medicine through mindfulness practices (Gotink et al., 2015).

While this review has focused on those populating the extremes of the social, behavioral, and cognitive spectrums, the question remains as to whether individuals within a cultural norm can alter their behavior to become more compassionate and display less selfish behavior. For this non-clinical cohort there exists an interesting non-clinical approach encompassed by mindfulness-based and compassion meditation. In one BOLD fMRI based study of expert and novice compassion meditators, an increase in activity was observed in the ACC, amygdala, and the insular cortex (among other regions) of expert meditators during meditation compared with non-meditative rest in response to negative emotional sounds (Lutz et al., 2008), indicating that compassion meditation increased activation of these emotion-processing limbic regions that are connected to the prefrontal cortex (also see Fox et al., 2016). These include regions implicated in the selfish–selfless spectrum as outlined previously in this text. The degree of activation of these regions also correlated with the self-reported depth of meditation and the degree of meditative training, indicating that additional experience activates these regions of the brain to a greater degree. Some groups have attempted to bring this practice to the clinical psychology setting (Fox et al., 2016). One study found that 12 weeks of mindfulness-based intervention with compassion meditation in a group with social anxiety disorder resulted in a significant improvement in social anxiety symptoms, depression, social adjustment, and enhancement of compassion, all compared to a control group that was placed on a waiting list at the beginning of the study (Koszycki et al., 2016).

In the general population, mindfulness-based practices have been reported to promote a plethora of effects on the whole body: reducing stress hormones, reducing inflammation, promoting pain relief and wound healing (Hofmann et al., 2011; Lutz et al., 2013; Zeidan et al., 2015; Rosenkranz et al., 2016). Other forms of meditation including concentrative eye-gazing and controlled breathing have been shown to result in voluntary activation of components of the sympathetic nervous system, changes in plasma catecholamine and serum cortisol concentrations, and an attenuation of the innate immune response as measured by plasma cytokines (Kox et al., 2014) and IL-6 markers (Pace et al., 2009). The study of altruism and psychopathy has implications beyond these traits, as similar brain regions are affected in Post-Traumatic Stress Disorder (Keding and Herringa, 2016; Rinne-Albers et al., 2017). Yoga and controlled eye movement therapies, called Eye Movement Desensitization and Reprocessing (EMDR), are techniques used for post-traumatic stress (Zepeda Méndez et al., 2018) and recognized by APA (2017) and United States Department of Veterans Affairs (2017). This suggests meditative or contemplative practices can affect acquired cognitive traits, including promoting unselfish behavior.

## Summary

Altruism as envisioned by Auguste Comte exists in the general population and in zealous altruists who anchor the benevolence end of the Selfish–Selfless Spectrum. Advances in genetics, psychology, and neurobiology have increased our understanding of social neurocircuitry in the human brain, providing critical insights into resolving ongoing philosophical, biological, and social debate over “universal selfishness” or “universal goodness” characterizing human behavior. Both positions are partial truths based on the components of the Selfish–Selfless Spectrum being observed. As a lens into the social brain, the extremes of the Selfish–Selfless Spectrum defined by callous, unemotional psychopaths and dynamic, zealous altruists reveal the importance of both genetic and neuron-based heredity systems and reward processes in strongly influencing actions toward others and cooperative behavior. Critically, individuals with genes associated with developing dangerous social disorders such as callous, unemotional traits have the potential to modify those conditions using cognitive therapeutic interventions (e.g., strong positive parenting, compassion training) to change where they reside on the social spectrum. Evidence from population groups ranging from incarcerated juveniles, adopted children, twins, and meditators point to the important role of neuroplasticity and reward learning circuitry in forming and reforming of neural connections that determine our behavior. Success with treatment programs promoting positive behavior via the brain reward system in these diverse groups suggests promise as a therapeutic approach to mitigating violent, destructive behavior. Approaches involving introspection and promoting acts of compassion that activate the reward system, such as mindfulness training, are entering the mainstream of clinical treatment for pain management, depression, stress, and behavior modification.

## Author Contributions

JS and DG contributed equally to the conception, design, writing of the manuscript, revisions, and approval of the submitted version.

## Conflict of Interest Statement

The authors declare that the research was conducted in the absence of any commercial or financial relationships that could be construed as a potential conflict of interest.
